# Relationship between hypertransmission defect size and progression in eyes with intermediate age-related macular degeneration

**DOI:** 10.1038/s41433-024-03338-0

**Published:** 2024-09-15

**Authors:** Onnisa Nanegrungsunk, Giulia Corradetti, Phichayut Phinyo, Janejit Choovuthayakorn, Srinivas R. Sadda

**Affiliations:** 1 Doheny Imaging Reading Center and Doheny Eye Institute, Pasadena, CA USA; 2 Department of Ophthalmology, David Geffen School of Medicine, University of California, Los Angeles, Los Angeles, CA USA; 3 Department of Ophthalmology, Faculty of Medicine, Chiang Mai University, Chiang Mai, Thailand; 4 Department of Family Medicine, Faculty of Medicine, Chiang Mai University, Chiang Mai, Thailand

**Keywords:** Predictive markers, Macular degeneration

## Abstract

**Objectives:**

To determine the associations between the presence of various-sized hypertransmission defects (hyperTDs) and progression to incomplete retinal pigment epithelial (RPE) and outer retinal atrophy (iRORA) and complete RORA (cRORA) in eyes with intermediate age-related macular degeneration (iAMD).

**Methods:**

Optical coherence tomography (OCT) data from consecutive iAMD patients, were retrospectively reviewed. All of iAMD eyes with or without iRORA (but not cRORA) at baseline were included. Graders evaluated the presence of hyperTDs at baseline (small: 63–124 µm; medium: 125–249 µm; large: ≥ 250 µm in diameter on choroidal en face OCT) and the progression two years later.

**Results:**

Of the 145 eyes that not developed neovascular AMD at two years, the eyes that progressed to or developed iRORA or cRORA included 13 eyes (10.7%), 5 eyes (83.3%), 9 eyes (81.8%), and 6 eyes (85.7%) in the groups with no, small, medium, and large hyperTDs at baseline, respectively (*P*-value < 0.001). The odds ratios (95% CI) for progression were 41.6 (4.5–383.6), 37.4 (7.3–192.0), and 49.9 (5.6–447.1) in the small, medium, and large hyperTDs groups, compared to no hyperTDs (*P*-value ≤ 0.001). Eyes with ≥ 2 hyperTDs also showed more frequent progression than eyes with one or no hyperTDs (100% vs. 16.4%; *P*-value < 0.001).

**Conclusions:**

While most iAMD eyes with no hyperTDs remained stable on OCT over two years, eyes with hyperTDs of any size appeared to be at a higher risk for progression. HyperTDs may provide an important OCT biomarker for identifying high-risk iAMD patients.

## Introduction

Geographic atrophy (GA) and exudative neovascular age-related macular degeneration (nAMD) are considered advanced forms of AMD and are leading causes of an irreversible blindness in the aging population [1]. While anti-vascular endothelial growth factor (anti-VEGF) has been a proven and effective therapy for nAMD for the past two decades, treatments for GA in the form of anti-complement agents (pegcetacoplan and avacincaptad pegol) have only recently become available [2–8]. Whereas significant visual improvement has been demonstrated with anti-VEGF treatment [3–6], treatments for GA only appear to modestly slow the decline, with vision loss continuing despite therapy [7–10]. These outcomes have suggested that earlier intervention prior to the onset of manifest atrophy may be necessary to achieve better functional outcomes. As a result, there has been considerable interest in identifying high-risk biomarkers prior to the development of GA, which may provide new targets for intervention.

Traditionally, GA has been defined as any sharp, round or oval area of hypopigmentation or depigmentation or apparent absence of retinal pigment epithelium (RPE) in which choroidal vessels are visible on a colour fundus photograph (CFP) [11]. Fundus autofluorescence (FAF) imaging has facilitated the detection and measurement of GA by providing high contrast between the borders of GA and the surrounding relatively intact retina; consequently, FAF imaging has become the mainstay technique for monitoring atrophy in clinical trials [7, 8, 12–14]. Optical coherence tomography (OCT) has significant advantages over planar imaging technologies in that it is depth-resolved, allowing discernment of which individual retinal layers are impacted by the atrophic process. The Classifications of Atrophy Meeting (CAM) proposed new terminology and definitions for atrophy on OCT, using the designation retinal pigment epithelium and outer retinal atrophy (RORA) for this purpose [15, 16]. Complete RORA (cRORA) was defined as a region of choroidal hypertransmission at least 250 µm, with a zone of overlying RPE attenuation or disruption also at least 250 µm, and evidence of overlying photoreceptor degeneration in the absence of a scrolled RPE or other signs of an RPE tear. Incomplete RORA (iRORA) was defined by similar component features as cRORA, but not fulfil the specific criteria of 250 µm required for cRORA [15, 16]. It should be acknowledged, that it is certainly possible that iRORA or cRORA may be manifest on OCT before GA becomes clearly visible on CFP or FAF [15, 16].

The true value of defining iRORA and cRORA on OCT is that may offer potential new biomarkers and endpoints to monitor the progression to atrophy in a more granular fashion. A challenge for the use of iRORA/cRORA is that they may require expert readers for reliable assessment, and some features such as the RPE disruption may be difficult to assess in the absence of high-quality scans [17]. To address these concerns, some researchers have suggested focusing on a single component of the iRORA/cRORA definition, a choroidal hypertransmission. With a choroidal en face image, the hypertransmission appears as discrete regions of hyperreflectivity (termed “hypertransmission defects or hyperTD”) which readily lend themselves reliable identification and quantification [18–21]. HyperTD ≥ 250 µm in diameter have been suggested to be irreversible or persistent, and have been proposed as a marker for the development of GA [20]. The fate of smaller hyperTD’s, however, is less certain and they may represent a reversible phenotype, which may be of particular relevance in selection subjects for interventional trials.

The reliability of assessing various-sized hyperTDs was reviewed in our recent report and showed an acceptable agreement (Gwet’s agreement coefficient: 0.89) between graders [21]. The prevalence of hyperTDs in eyes with intermediate AMD (iAMD) was 16.8% and the rates of persistence were 100.0% for large (*≥ *250 µm) hyperTDs, 72.7% for medium (125–249 µm) hyperTDs, and 53.3% for small (63–124 µm) hyperTDs over two years [21]. The progression of these various-sized hyperTDs to more advanced stages of AMD or their resolution has not been well-described. In this study, we characterize the association between the various sizes of the hyperTDs in eyes with iAMD and their progression to iRORA, cRORA, and nAMD over a two-year period.

## Methods

This retrospective study was approved by the Institutional Review Board of the University of California, Los Angeles (UCLA) and a waiver of informed consent was granted. The research was conducted and conformed with the Declaration of Helsinki and the Health Insurance Portability and Accountability Act of 1996.

### Study population, and inclusion and exclusion criteria

The study design and eligibility criteria for this study were described in our previous report on the prevalence and persistence of various-sized hyperTDs [21]. In brief, consecutive iAMD patients who had the first available OCT images (Cirrus spectral domain OCT; Carl Zeiss Meditec, CA, USA) obtained at the Doheny - UCLA Eye Center retina clinic between January and February 2017 (baseline visit) were retrospectively identified. Individuals who were at least 50 years old with at least one large druse (basal width >125 µm) within two-disc diameter from the foveal centre on the OCT in either eye were identified as having iAMD [22, 23]. This assessment was performed by a certified Doheny Image Reading and Research Lab (DIRRL) grader (O.N.). Eyes with advanced AMD, signs of non-exudative nAMD (e.g., double-layer sign [DLS]) [24–26], other coexisting retinal pathology affecting retina or choroid (e.g., diabetic macular oedema, pathologic myopia, etc.), or eyes with ungradable OCT images were excluded. Advanced AMD was defined by the presence of exudative macular neovascularization (MNV) or by the presence of cRORA in the posterior pole. MNV was recognized in accordance with the Consensus on Neovascular AMD Nomenclature (CONAN) criteria for identifying MNV on structural OCT [27]. Exudative MNV was recognized by the presence of fluid in the intraretinal, subretinal, or sub-RPE space on OCT and/or haemorrhage, thought to be related to the presence of MNV. cRORA and iRORA were recognized in accordance with the CAM definitions as previously mentioned above [15, 16]. A thick DLS was defined by an area of shallow and irregular elevation of the RPE from Bruch’s membrane with non-homogenous sub-RPE reflectivity [24, 26], and is thought to correspond to non-exudative type 1 MNV. If both eyes were eligible, then both eyes were included in this study.

Due to the retrospective and observational design, each patient was individually followed and imaged according to clinical stage, disease activity, and practice patterns of the managing physician. The intervals between clinic visits and the frequency of imaging consequently varied among patients, ranging from two weeks to once a year. As 93.1% of iRORA lesions were noted to have converted to cRORA within 24 months in a prior study [28], only iAMD eyes with two-year (*± *3 months) follow-up OCT images were included in the longitudinal analyses.

### Image acquisition

All eligible iAMD eyes had Cirrus SD-OCT imaging performed using a 6 × 6-mm macular cube scan (512 ×128) protocol centred on the fovea, which consisted of 128 horizontal rasters with 512 A-scans per B-scan, yielding a 47-µm space between adjacent horizontal scans. To produce an en face OCT image, a custom choroidal slab 64–400 µm below Bruch’s membrane was generated using the instrument’s automated segmentation software.

### Grading procedure

After the graders (O.N. and G.C.) were trained and demonstrated consistent grading in the training exercise, they independently evaluated the en face OCT images at the baseline and follow-up. In accordance with previous studies [20, 22], a hyperTD was identified as a well-demarcated bright region seen on the en face image with evidence of at least some RPE alterations on the corresponding B-scans that explained the hypertransmission. In some cases, hyperTDs appeared as a ring of hypertransmission with a central core of hypotransmission (“donut”) due to a thickened RPE or other overlying material in this area [20]. More than one hyperTD could be identified in each study eye. Graders identified the hyperTDs, measured their greatest linear dimensions (GLDs) in any axis on the en face image using the instrument’s calliper tool, counted the number of hyperTDs, and evaluated the overlying photoreceptor on the corresponding horizontal B-scan for each hyperTD lesion, to determine which hyperTDs would also meet criteria for iRORA. All baseline hyperTDs were tracked and evaluated at the two-year (*± *3 months) follow-up timepoint using the same grading protocol implemented at baseline. All available OCT images from baseline to the final follow-up visit were also assessed for any evidence of exudative MNV or thick DLS (e.g., nonexudative MNV) in order to classify the nAMD progression. Once the study eye was identified to have any sign of nAMD, that eye was not evaluated any further for hyperTDs, iRORA, or cRORA due to the appearance of exudation, which could affect the reflectivity and assessment of hypertransmission, and thus resulted in exclusion from the primary analysis.

As there are no specific prior guidelines for classifying hyperTD size, this study adopted conventions used for assessing drusen size. The hyperTDs were categorized into three categories based on their maximum GLD on the en face OCT: small (63–124 µm), medium (125–249 µm), and large (250 µm or more).

Disagreements between graders on any lesion or visit were adjudicated by the senior DIRRL investigator (S.R.S), who provided the final consensus in such cases.

### Changes over two years: stable, progression, or regression

As this study was a novel and exploratory analysis, to our knowledge, there are no established conventions to define stability, progression, and regression in the context of hyperTDs. Thus, we introduced new definitions for the purpose of our analyses (Table 1). In brief, “stable” was defined to encompass: 1) the continuing absence of a hyperTD, 2) the presence of hyperTD with or without iRORA similar to the baseline, or 3) the presence of a new hyperTD without iRORA or cRORA at the follow-up visit. “Progression” was defined as: 1) the conversion of the baseline hyperTD lesion to iRORA, cRORA, exudative MNV, or thick DLS, 2) the new presentation of hyperTD that already met criteria for iRORA or cRORA, or 3) the presence of exudative MNV or thick DLS. “Regression” was defined as the absence of hyperTDs or absence of iRORA (if noted at baseline) on the follow-up OCT image.

**Table 1:** Definitions of hyperTD-related changes at 2-year follow-up in eyes with intermediate AMD.

Baseline visit		Follow-up visit					Terms
		No hyperTD	HyperTD presence^a^				
			No iRORA	iRORA presence	cRORA presence	Exudative MNV or thick DLS presence	
No hyperTD		X	X				Stable
HyperTD presence	No iRORA		X				
	iRORA presence			X			
No hyperTD				X	X	X	Progression
HyperTD presence	No iRORA			X	X	X	
	iRORA presence				X	X	
No hyperTD		NA					Regression
HyperTD presence	No iRORA	X					
	iRORA presence	X	X				

At least one progression or regression event of a lesion was deemed a change. If both progressed and regressed lesions were present in the same eye, the eye was deemed to have progressed.

*cRORA* complete retinal pigment epithelial and outer retinal atrophy, *DLS* double layer sign, *hyperTD* hypertransmission defect, *iRORA* incomplete retinal pigment epithelial and outer retinal atrophy, *MNV* macular neovascularization, *NA* not available

^a^Regardless of changes in the size categories of hyperTD.

At least one progression or regression event of a lesion was deemed a “change” in that eye for eye-level analysis. If both progression and regression events occurred in the same eye, the eye was deemed to have progressed. If stable lesions and progressed or regressed lesions were present in the same eye, the eye was deemed to have progression or regression. If there were stable, progressed and regressed lesions all present in the same eye, the eye was deemed to have progressed. Thus, a hierarchical approach was used to weight individual lesions events for eye-level classification with progressed > regressed > stable.

The primary analysis focused on eyes that progressed to or developed iRORA or cRORA (only non-neovascular AMD), but the secondary analysis included all progression events to iRORA, cRORA, and nAMD.

### Statistical analysis

The study eyes and/or patients were divided into four groups according to the presence and size of hyperTDs at baseline: no hyperTDs, small hyperTDs, medium hyperTDs, and large hyperTDs groups. If two or more lesions were present in the eye, the largest hyperTD lesion was selected to represent the overall hyperTD size in that eye. At the patient level, if two or more lesions were present in that individual (from either one or two eyes), the patient’s largest hyperTD lesion was selected to represent the overall hyperTD size. The descriptive statistics of the categorical data were described based on frequency (percentage) and compared with Fisher’s exact test. The generalized estimating equation (GEE) was used to determine the univariable odds ratios (ORs) of the associations between the hyperTD and progression at eye level. A P-value of less than 0.05 was considered statistically significant. All statistical analysis was performed with Stata 17 (StataCorp, College Station, TX, USA).

## Results

### Baseline characteristics

A total of 247 consecutive iAMD patients (273 eyes) who met the eligibility criteria were identified. Two-year (*± *3 months) follow-up OCT data were available for 177 eyes (64.8%) of 171 patients (69.2%), which were not statistically significant between groups (*P*-value *=* 0.812).

After excluding 32 eyes that developed nAMD during the follow-up, 145 eyes (140 patients) remained in the non-neovascular AMD category and were thus included in the primary analysis. Of these 145 eyes, 121 eyes (83.4%) had no hyperTDs at baseline. Among the 24 eyes with hyperTDs, 11 eyes (45.8%) had medium-sized hyperTDs and 13 eyes (54.2%) had a single hyperTD lesion. The minimum and maximum numbers of lesions per eye were 1 and 5. The median (interquartile range) number of hyperTDs lesions per eye was 1 (1, 2.5). The details of the baseline characteristics of non-neovascular AMD eyes in each hyperTD category are shown in Table 2.

**Table 2:** Baseline characteristics of all eyes that retained non-neovascular AMD at 2-year follow-up.

Factors	No hyperTD (*n* = 116 patients)	Small hyperTD (*n* = 6 patients)	Medium hyperTD (*n* = 11 patients)	Large hyperTD (*n* = 7 patients)	*P*-value
Female, No. (%)	68 (58.6)	3 (50.0)	3 (27.3)	2 (28.6)	0.108
Age, year (SD)	79.5 (8.5)	83.2 (11.0)	77.9 (10.0)	81.2 (6.4)	0.631
	**No hyperTD (*n*** = **121 eyes)**	**Small hyperTD (*n*** = **6 eyes)**	**Medium hyperTD (*n*** = **11 eyes)**	**Large hyperTD (*n*** = **7 eyes)**	
Presence of baseline iRORA, No. (%)	NA	5 (83.3)	9 (81.8)	7 (100.0)	0.134^a^

*GLD* greatest linear dimension, *hyperTD* hypertransmission defect, *iRORA* incomplete retinal pigment epithelial and outer retinal atrophy, *large hyperTD* GLD ≥ 250 µm, *medium hyperTD* GLD 125–249 µm, *NA* not available, *No.* number, *SD* standard deviation, *small hyperTD* GLD 63–124 µm.

^a^Comparison among hyperTD groups.

### Changes in eyes that remained in non-neovascular AMD over two years

**Fig. 1:**
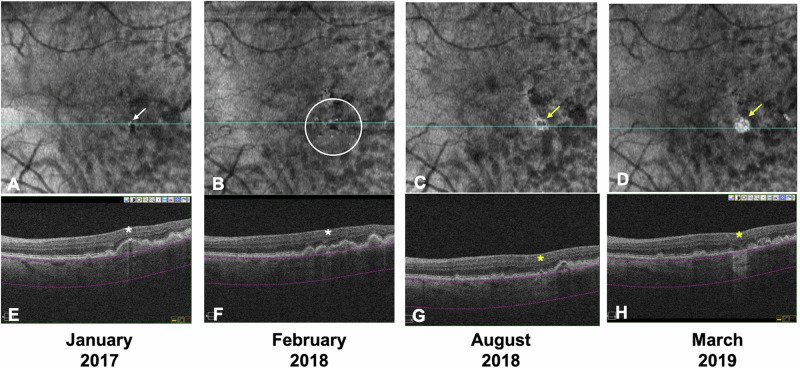
An example of eye with hyperTD with progression. A single small hyperTD (white arrow) is detected on the baseline en face OCT (**A**). The corresponding horizontal OCT B-scan (**E**) shows choroidal hypertransmission and a small region of RPE disruption, but no overlying photoreceptor degeneration (white asterisk). The criteria for iRORA are not met at baseline on OCT. Fourteen months from baseline, multiple small hyperTDs (white circle) are evident with a progression to iRORA (**B** and **F**). Six months later (20 months from baseline), these small hyperTDs coalesced and developed into large hyperTDs with a hyporeflective core (donut; yellow arrow) on the en face OCT (**C**) and iRORA (yellow asterisk) on the corresponding B-scan (**G**). At the final follow-up (26 months from baseline), a large hyperTD without a hyporeflective core (yellow arrow) is evident on the en face OCT (**D**) with cRORA (yellow asterisk) on the corresponding B-scan (**H**). This eye is deemed a progression eye.

**Fig. 2:**
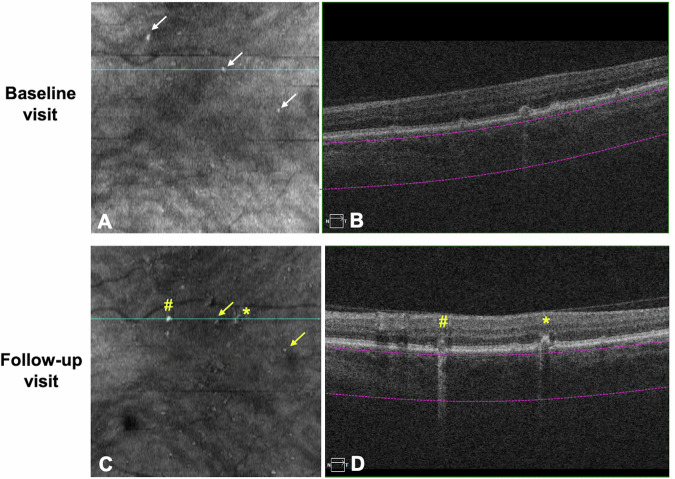
An example of eye with hyperTDs with progression. Three medium hyperTDs (white arrows) are detected at baseline on the en face OCT (**A**). None of the hyperTD lesions meet the criteria for iRORA and one of the lesions shows in **B**. At the follow-up visit, two of the baseline hyperTDs persisted (yellow arrows) on the en face OCT (**C**). Two new hyperTDs (yellow sharp and yellow asterisk) are also noted on the en face OCT (**C**) with iRORA (yellow sharp and yellow asterisk) on the B-scan (**D**). This eye is deemed a progression eye.

Among 121 eyes with no hyperTDs at baseline, 108 eyes (89.3%) remained stable, and 13 eyes (10.7%) progressed to or developed iRORA or cRORA. Among 24 eyes with hyperTDs at baseline, 2 eyes (8.3%) remained stable, 20 eyes (83.3%) progressed, and 2 eyes (8.3%) regressed. Details of the changes in each hyperTD category are shown in Table 3, which illustrates that the changes differed significantly among these groups (*P*-value *<* 0.001). A univariable OR showed significant associations between each hyperTD’s size and progression. The ORs (95% confidence interval [CI]) were 41.6 (95% CI: 4.5–383.6; *P*-value *=* 0.001) in the small hyperTDs group, 37.4 (95% CI: 7.3–192.0; *P*-value *<* 0.001) in the medium hyperTDs group, and 49.9 (95% CI: 5.6–447.1; *P*-value *<* 0.001) in the large hyperTDs group, compared to no hyperTDs at baseline group. The presence of baseline iRORA was also associated with progression (OR: 43.7; 95% CI: 11.5–166.0; *P*-value *<* 0.001). Regardless of size, a number of hyperTD lesion per eye was also associated with progression: 100% of eyes with two or more hyperTD lesions progressed versus 16.4% of eyes with one or no hyperTDs (*P*-value *<* 0.001). Two eyes that showed regression had a single hyperTD lesion at baseline. One eye demonstrated the disappearance of baseline small hyperTD and another eye demonstrated the disappearance of baseline iRORA. Examples of eyes with hyperTDs that progressed to or developed iRORA or cRORA are shown in Figs. 1 and 2.

**Table 3:** Changes in non-neovascular AMD eyes during 2-year follow-up.

	No hyperTD (*n* = 121 eyes)	Small hyperTD (*n* = 6 eyes)	Medium hyperTD (*n* = 11 eyes)	Large hyperTD (n = 7 eyes)	*P*-value
Stable, No. (%)	108 (89.3)	0 (0)	1 (9.1)	1 (14.3)	<0.001
Progression to or development of iRORA or cRORA, No. (%)	13 (10.7)	5 (83.3)	9 (81.8)	6 (85.7)	
Regression, No. (%)	NA	1 (16.7)	1 (9.1)	0 (0)	

*cRORA* complete retinal pigment epithelial and outer retinal atrophy, *GLD* greatest linear dimension, *hyperTD* hypertransmission defect, *iRORA* incomplete retinal pigment epithelial and outer retinal atrophy, *large hyperTD* GLD ≥ 250 µm, *medium hyperTD* GLD 125–249 µm, *NA* not available, *No.* number, *small hyperTD* GLD 63–124 µm.

### Overall changes of all iAMD eyes over two years (both neovascular and non-neovascular AMD)

Most of the eyes with no hyperTDs (108 eyes; 74.5%) remained stable over two years, while most of the eyes with hyperTDs (28 eyes; 87.5%) progressed. The number of eyes that progressed to or developed iRORA, cRORA, or nAMD were 37 eyes (25.5%), 8 eyes (88.9%), 11 eyes (84.6%), and 9 eyes (90.0%) in the groups with no hyperTDs, small hyperTDs, medium hyperTDs and large hyperTDs, respectively (*P*-value *<* 0.001). Compared to no hyperTDs group, the univariable ORs (95% CI) for progression were 23.5 (95% CI: 2.8–193.8; *P*-value *=* 0.003), 16.1 (95% CI: 3.4–76.1; *P*-value *<* 0.001), and 26.4 (95% CI: 3.2–215.3; *P*-value *=* 0.002), in small hyperTDs, medium hyperTDs and large hyperTDs groups, respectively. The presence of baseline iRORA was also associated with progression (OR: 20.0; 95% CI: 5.7–70.0; *P*-value *<* 0.001).

## Discussion

As hyperTD is a relatively new OCT feature, this study describes the natural courses of various-sized hyperTDs in iAMD eyes and the association of hyperTDs with progression to more advanced stages of AMD. Over two years of follow-up, the majority of eyes with no hyperTDs at baseline (74.5%) were stable while eyes with hyperTDs (87.5%) were more likely to progress (ORs ranged from 16.1 to 26.4; *P*-value *<* 0.005), implying that a hyperTD of any size may be a biomarker for progression to both neovascular and non-neovascular AMD.

To compare with other previous studies, and because choroidal hypertransmission is a component of OCT definitions of atrophy, we performed the primary analysis on eyes that remained at the non-neovascular AMD stage. Similar to the full-dataset analysis, most eyes without hyperTDs (89.3%) remained stable, while most eyes with hyperTDs (83.3%) progressed to iRORA or cRORA. Shi et al. reported an association between hyperTDs ≥ 250 µm (OR: 14.5; 95% CI: 4.8–54.0; *P*-value *<* 0.001) and nascent GA [22]. Laiginhas et al. also reported that hyperTDs ≥ 250 µm increased the risk of GA formation by 80.4 times (95% CI: 10.7–614; *P*-value *<* 0.001) [20]. Although different in methods and statistical analysis, the association of large hyperTDs (*≥ *250 µm) and the progression to atrophy were similar to our study (OR: 49.9; 95% CI: 5.6–447.1; *P*-value *<* 0.001). Our study also demonstrated the association of small and medium hyperTDs with progression to iRORA or cRORA. Although the 95% CIs were relatively wide in our study and the ORs did not increase with larger hyperTD size, the identification of these associations still signifies a meaningful trend. HyperTDs evident on en face OCT, even small or medium-sized lesions, reflect transmitted light through a defect or irregularity of the RPE, which then presumably further progresses into a more advanced stage such as iRORA and cRORA. The problem of only considering large lesions (size ≥ 250 µm) is that many of these lesions may have already met the criteria for cRORA at the time of identification [29], as well as a point-of-no-return of lost visual acuity or irreversible atrophic structure. Small to medium-sized hyperTDs (*< *250 µm) are more attractive biomarkers as they may represent an earlier or precursor stage, but yet they are at substantially higher risk for progression to iRORA/cRORA and thus the frequency of their conversion could be evaluated in an early intervention trial.

In addition to size, the number of hyperTD lesions per eye (2 or more lesions per eye) appeared to be an important risk factor associated with progression in our study (*P*-value *<* 0.001). A possible explanation is that having multiple hyperTDs in an eye, irrespective of their size, may reflect more wide-spread or extensive disease and thus these eyes are more susceptible for progression. Based on our observations, we propose that the presence of small to medium-sized hyperTDs and a higher overall number of hyperTD lesions in an eye may be used to identify an eye that is high risk for progression and for inclusion in early therapeutic trials.

The presence of iRORA was also associated with progression to advanced AMD (*P*-value *<* 0.001). It should be noted that in prior studies, various other features (e.g., hyperreflective foci, hyporeflective drusen cores [hDC], subretinal drusenoid deposit [SDD], high-central drusen volume [≥ 0.03 mm^3^], thin DLS, cRORA in the fellow eye), have also been reported as risk factors for progression [20, 26, 30]. Due to the limited number of eyes in our study, we were not able to consider or control for these other previously described risk factors in the present analysis. It is possible that hyperTD may not have remained as an independent predictor of progression if these other factors were considered in the analysis. Future longitudinal studies with a larger number eyes, may allow the importance of hyperTD relative to these other risk factors to be more precisely assessed.

In other studies, the persistence of hyperTD has been shown to be size dependent [21, 22, 31]. Our previous report demonstrated that 53.3% of small hyperTDs and 72.7% of medium hyperTDs persisted, in contrast to 100.0% of large hyperTDs [21]. In this study, two of 32 eyes with hyperTDs regressed, one in small hyperTDs group and another in medium hyperTDs group. We speculate that with smaller hyperTD’s it may be possible for RPE cells to spread and “fill” the gap in a regenerative or compensatory process [21, 22]. If regression can truly occur this may be another attractive attribute for considering inclusion of small/medium-sized hyperTDs in early intervention trials. It is also possible that disappearance of small lesions could be an artifact of the image acquisition protocol. While we would not expect even small hyperTDs to completely disappear between the 47-µm inter-scan space, however, they may have been more difficult to discern depending on how they were positioned relative to the scans.

The retrospective design and the relatively small number of eyes account for the main limitations of the present study. In addition, our analyses were limited to a single OCT device, and it is not clear how our results would apply to other devices. However, previous reports have shown that iRORA/cRORA lesions may be detected reliably on both Cirrus and Spectralis images [32], and that hyperTDs can be detected on Spectralis or Swept-source OCT en face images as well [20, 33, 34]. We did not have corresponding visual function data to describe the functional deficit associated with the hyperTD lesions. Such information will ultimately be required if hyperTDs are to be used in early intervention trials. Our study also entirely relied on structural OCT and did not consider other modalities. The influences of other confounding factors (e.g., hyperreflective foci, SDD and hDC) for AMD progression or cRORA development along with hyperTD were not included in our study. Finally, our analysis was limited to two years, and thus we cannot assess the relevance of hyperTDs to conversions at longer or shorter intervals.

Despite these limitations, our study has several strengths. These include a consistent OCT scanning protocol across all subjects and the use of reading center graders who have demonstrated a high-level of reproducibility from grading various-sized hyperTDs in previous reports [21].

In summary, while most of the iAMD eyes without hyperTDs at baseline are likely to remain stable over the next two years, eyes with hyperTDs of any size, especially if there are multiple hyperTDs, are at substantially higher risks for progression to iRORA, cRORA, and/or nAMD. Thus, hyperTDs may be useful for risk stratification and selection of high-risk patients for early intervention clinical trials for iAMD.

## Summary

### What was known before

- HyperTDs size 250 micron or greater have been suggested to be irreversible or persistent, and have been proposed as a marker for the development of geographic atrophy.

### What this study adds

- HyperTDs of any size, especially if there are multiple hyperTDs, are at substantially higher risks for progression to iRORA, cRORA, and/or neovascular AMD. Thus, hyperTDs may be useful for risk stratification and selection of high-risk patients for early intervention clinical trials for intermediate AMD.

## Data Availability

The data supporting the findings of this study are not publicly accessible due to privacy concerns and can be available from the corresponding author upon reasonable request.
